# Supplementary material for: Conscious perception of natural images is constrained by category-related visual features
Source: Nat Commun. 2019 Sep 11;10:4106. doi: 10.1038/s41467-019-12135-3 (PMC6739304; doi:10.1038/s41467-019-12135-3)
Supplement: Supplementary file 2 — Reporting Summary [file 41467_2019_12135_MOESM2_ESM.pdf]

nature research

Corresponding author(s): Daniel Lindh, p.j.d.lindh@uva.nl

Last updated by author(s): Jul 19, 2019

Reporting Summary

Nature Research wishes to improve the reproducibility of the work that we publish. This form provides structure for consistency and transparency in reporting. For further information on Nature Research policies, see Authors & Referees and the Editorial Policy Checklist.

Statistics

For all statistical analyses, confirm that the following items are present in the figure legend, table legend, main text, or Methods section.

n/a	Confirmed	
☐	☒	The exact sample size (*n*) for each experimental group/condition, given as a discrete number and unit of measurement
☐	☒	A statement on whether measurements were taken from distinct samples or whether the same sample was measured repeatedly
☐	☒	The statistical test(s) used AND whether they are one- or two-sided<br>*Only common tests should be described solely by name; describe more complex techniques in the Methods section.*
☒	☐	A description of all covariates tested
☐	☒	A description of any assumptions or corrections, such as tests of normality and adjustment for multiple comparisons
☐	☒	A full description of the statistical parameters including central tendency (e.g. means) or other basic estimates (e.g. regression coefficient) AND variation (e.g. standard deviation) or associated estimates of uncertainty (e.g. confidence intervals)
☐	☒	For null hypothesis testing, the test statistic (e.g. *F*, *t*, *r*) with confidence intervals, effect sizes, degrees of freedom and *P* value noted<br>*Give P values as exact values whenever suitable.*
☒	☐	For Bayesian analysis, information on the choice of priors and Markov chain Monte Carlo settings
☐	☒	For hierarchical and complex designs, identification of the appropriate level for tests and full reporting of outcomes
☐	☒	Estimates of effect sizes (e.g. Cohen's *d*, Pearson's *r*), indicating how they were calculated

Our web collection on statistics for biologists contains articles on many of the points above.

Software and code

Policy information about availability of computer code

Data collection	Data was collected using psychtoolbox (version 3: http://psychtoolbox.org/) in MatLab 2016b (Mathworks) for presenting stimuli and collecting keyboard reponses.
Data analysis	Data was analyzed in python 3.6 using Sci-kit learn (0.20.2), caffe deep learning framework, and SciPy (v 1.1.0). See below for code and data availability.

For manuscripts utilizing custom algorithms or software that are central to the research but not yet described in published literature, software must be made available to editors/reviewers. We strongly encourage code deposition in a community repository (e.g. GitHub). See the Nature Research guidelines for submitting code & software for further information.

Data

Policy information about availability of data

All manuscripts must include a data availability statement. This statement should provide the following information, where applicable:

- Accession codes, unique identifiers, or web links for publicly available datasets
- A list of figures that have associated raw data
- A description of any restrictions on data availability

Data and code are available at: https://github.com/Charestlab/abdcnn

Field-specific reporting

Please select the one below that is the best fit for your research. If you are not sure, read the appropriate sections before making your selection.

☐ Life sciences ☒ Behavioural & social sciences ☐ Ecological, evolutionary & environmental sciences

Behavioural & social sciences study design

All studies must disclose on these points even when the disclosure is negative.

Study description	In this study we collected quantitative behavior during a well-known attentional task (Attentional Blink). We based our analyses on the performance (hit or miss) of the two targets for each trial.
Research sample	In the first experiment, twenty participants (undergraduate students, 19 females; age mean = 20.1 ± 1.2) with normal, or corrected-to-normal, vision were recruited from the University of Birmingham's School of Psychology's research participation scheme (RPS).<br>In the second experiment, 24 participants were recruited (19 females; age mean: 19.38 ± 0.95), with normal, or corrected-to-normal, vision.<br>Our sample contains a clear over representation of female participants (as is most often the case when in participation schemes in psychology departments), but we are not aware of evidence for a difference between sexes in a perceptual task like the attentional blink, and comparing sexes is beyond the scope of this manuscript.
Sampling strategy	Participants were recruited via the research participation scheme at the University of Birmingham's School of Psychology (convenience sampling). A sample size of 20 participants was chosen, based on previous literature on the attentional blink.
Data collection	During each session, participants were comfortably seated in dim lit testing cubicles at the School of Psychology. Stimulation was presented using a Stone desktop PC (Stone PC Pro), using the psychophysical toolbox in Matlab on a windows 10 operating system, and a 22 inch LCD monitor (60hz refresh rate; iiyama prolite b2283hs). The experimenter greeted the participant, and started the stimulation paradigm, before leaving the room. Instructions were presented on the computer screen and read by the participants before starting the task. The experimenter was seated outside of the testing cubicle. The experimenter was aware of the conditions and hypotheses during data collection, but could not influence participants in their responses as instructions were presented as part of the stimulation protocol in psychtoolbox, and read by the participant. The experimenter could answer questions about the task instructions.
Timing	Data for the first experiment was collected through November 2016 and January 2017. Data for the experiment was collected between November 2017 and February 2018.
Data exclusions	In the first study, three participants were excluded because of incomplete data collection and technical errors. No data were excluded in the second experiment.
Non-participation	No participants dropped out during testing.
Randomization	Participants were not allocated to different experimental groups

Reporting for specific materials, systems and methods

We require information from authors about some types of materials, experimental systems and methods used in many studies. Here, indicate whether each material, system or method listed is relevant to your study. If you are not sure if a list item applies to your research, read the appropriate section before selecting a response.

Materials & experimental systems

n/a	Involved in the study	
☒	☐	Antibodies
☒	☐	Eukaryotic cell lines
☒	☐	Palaeontology
☒	☐	Animals and other organisms
☐	☒	Human research participants
☒	☐	Clinical data

Methods

n/a	Involved in the study	
☒	☐	ChIP-seq
☒	☐	Flow cytometry
☒	☐	MRI-based neuroimaging

Human research participants

Policy information about studies involving human research participants

Population characteristics	See above
Recruitment	See sampling strategy above for recruitment procedure. We cannot identify any biases except for an over representation of female participants. However, as discussed, we do not know of any reasons as to why this would change our results.
Ethics oversight	Both studies were reviewed and approved by the University of Birmingham ethical committee.

Note that full information on the approval of the study protocol must also be provided in the manuscript.
